# A single serving of mixed spices alters gut microflora composition: a dose–response randomised trial

**DOI:** 10.1038/s41598-021-90453-7

**Published:** 2021-05-28

**Authors:** Wei Wei Thwe Khine, Sumanto Haldar, Shou De Loi, Yuan-Kun Lee

**Affiliations:** 1 Department of Microbiology & Immunology, Yong Loo Lin School of Medicine, National University of Singapore, 5 Science Drive 2, Singapore, 117545 Singapore; 2 Functional Foods Forum, Faculty of Medicine, University of Turku, 20014 Turku, Finland; 3 Clinical Nutrition Research Centre (CNRC), Singapore Institute of Food and Biotechnology Innovations (SIFBI), Agency for Science Technology and Research (A*STAR), 14 Medical Drive, Singapore, 117599 Singapore; 4 Department of Surgery, National University Hospital, Tower Block, 1E Kent Ridge Road, Singapore, 119228 Singapore

**Keywords:** Microbiology, Molecular biology, Preclinical research

## Abstract

Short-term changes in dietary intake can induce changes in gut microbiome. While various dietary polyphenols have been shown to modulate gut microflora, the acute influence of polyphenol-rich mixed spices has not been explored in a controlled setting. We investigated the effects of a single serving of mixed spices Indian curry consumption, in two separate doses, on the gut microbiome in 15 healthy, Singaporean Chinese males, with age and BMI of 23.5 ± 2.4 years and 22.9 ± 2.2 kg/m^2^ respectively. We found that a low-polyphenol, no spices Dose 0 Control (D0C) meal led to an increase in *Bacteroides* and a decrease in *Bifidobacterium*. In comparison to D0C, there was significant suppression of *Bacteroides* (p < 0.05) and an increase in *Bifidobacterium* (p < 0.05) with increasing doses of curry meal Dose 1 Curry (D1C) and Dose 2 Curry (D2C) containing 6 g and 12 g mixed spices respectively. Significant correlations were also found between bacterial changes and plasma phenolic acids. No differences between treatments were observed in the alpha-diversity of the gut microflora. This study has shown that a single serving of mixed spices can significantly modify/restore certain commensal microbes, particularly in people who do not regularly consume these spices.

## Introduction

The gut microbiome has been widely acknowledged as a dynamic and complex community of bacteria that is dependent on extrinsic and intrinsic factors, of which diet is one of the most dominant modulators^1–3^. Several previous studies have shown that dietary patterns can affect gut microbiome^4–7^. Numerous studies indicate fruits^8–10^ and/or vegetables^11–14^ as well as whole grain cereals^12,15^ can directly affect gut microflora composition. Besides these dietary patterns, foods and established nutrients, various ‘non-nutrient bioactive compounds’ in diet, including polyphenols, can significantly modulate gut microflora composition^16–18^. Spices that are often used as culinary ingredients for flavouring, colouring or prolonging food shelf-life^19^ are rich in polyphenols that are generally poorly absorbed by the upper intestine and present in relatively high concentrations in the lower gut^20^. Therefore, they are expected to interact and modulate the gut microbiome.

Depending on the polyphenol type, they may either have antimicrobial effects towards certain gut bacteria, whereas, stimulatory effects on other types of bacteria^21,22^, as well as having the ability to alter the ratios of *Firmicutes/Bacteroidetes*^23^ and *Prevotella*/*Bacteroides*^24^*.* These studies highlighted the potential modulatory role of polyphenol-rich spices on the composition of the human gut microbiome. Given that nowadays, spices are widely consumed globally, albeit on an occasional basis, it is therefore important to investigate the acute effects of spice consumption on the gut microbiome. Most previous research examined the effect of single spices or polyphenolic extracts from single spices such as curcumin on both human subjects and in animal studies^18,21,25,26^. However, few prior studies have examined the effects of introducing a dietary concoction of several spices in combination, which is how they are typically consumed in different curries across various cultures worldwide. To the best of our knowledge, only one recent study has attempted to test the effects of five different mixed spices in combination (cinnamon, oregano, ginger, rosemary, black pepper and cayenne pepper), in dietary doses, on gut microbiome and found that up to 26 operational taxonomic units (OTUs) were modulated as a result of the mixed-spice treatment compared with placebo^27^.

Therefore, we investigated the acute effects on gut microflora of a combination of 7 different spices as a secondary analysis of a recently completed study that measured various metabolic outcomes^28,29^. Considering that this study was undertaken in an acute setting with two separate mixed spices doses, it provided an ideal setting to investigate acute, dose-dependent changes in gut microbiome as a result of the consumption of these mixed spices. From this same study, since we have previously reported dose-dependent increases in two phenolic acids (i.e., cinnamic acid (CNA) and phenylacetic acid (PAA)) as well as increase in the total polyphenol excretion (TPE)^29,30^, we have also investigated the associations between these parameters as objective markers of the spices intake with the changes in the gut microbiome.

## Results

### Basic demographics and dietary intake 3-days prior to each session

The mean age and BMI of the participants at the first session who provided the stool samples were 23.5 ± 2.4 years and 22.9 ± 2.2 kg/m^2^ respectively. The reported intakes of energy and some key nutrients during the 3-day ‘run-in periods’ immediately prior to the main study day (Day 1) are shown in Table 1. There were no significant differences between the intervention sessions in any of the nutrients or energy intake.

**Table 1:** Mean daily intake of energy and nutrients during the run-in periods prior to each intervention session.

Measurement	Mean ± SD			p value (one-way ANOVA; mixed-effects analysis)
	Dose 0 control (D0C) (n = 14)	Dose 1 curry (D1C) (n = 15)	Dose 2 curry (D2C) (n = 15)	
Energy (kcal)	1763.600 ± 377.666	1572.576 ± 429.014	1560.602 ± 406.569	0.1384
Carbohydrates (g)	189.385 ± 38.211	179.072 ± 51.176	183.486 ± 78.424	0.8262
Protein (g)	88.338 ± 27.345	78.561 ± 19.003	76.277 ± 20.264	0.1771
Total fats (g)	67.668 ± 19.673	57.709 ± 20.035	59.500 ± 20.667	0.2109
Saturated fats (g)	27.000 ± 9.586	23.832 ± 8.562	24.982 ± 7.659	0.5338
Fibre (g)	15.726 ± 6.151	13.969 ± 6.380	15.566 ± 7.358	0.417
Cholesterol (mg)	364.247 ± 165.379	301.156 ± 119.466	333.020 ± 162.609	0.2956
Sodium (mg)	3191.363 ± 766.049	2910.272 ± 861.957	3163.564 ± 1007.169	0.5686

P values were calculated from the statistical test of one-way ANOVA based on the consumption of each nutrient. D0C = Meal with no spices intervention, D1C = Meal with low (6 g) mixed spices intervention, D2C = Meal with high (12 g) mixed spices intervention, n = 15 each intervention, except for D0C, where the data from 1 subject was excluded because of being an outlier (extreme over-reporting).

### Changes in microbiome composition

**Figure 1:**
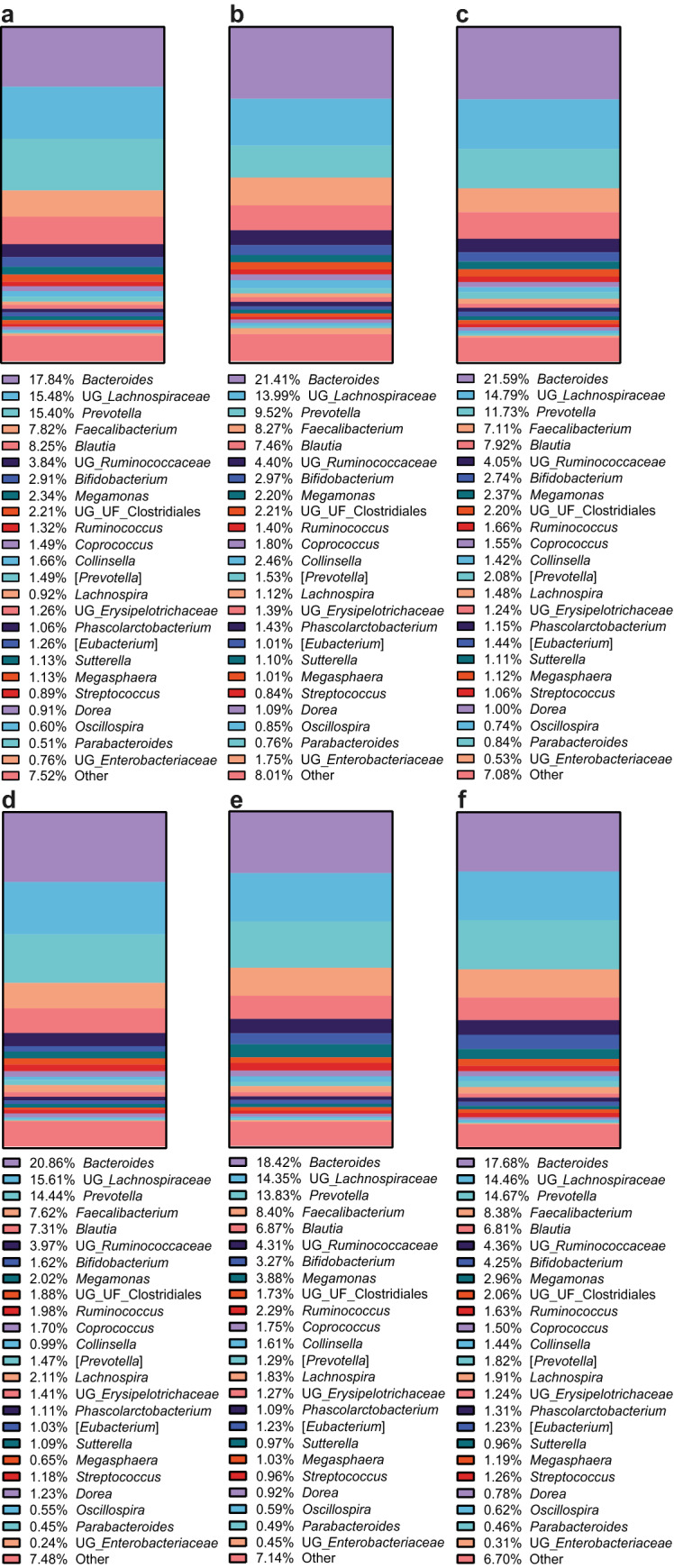
Comparison of the relative abundance of bacterial genera for the three different doses of spices intervention at Day 0 (baseline) (a-c) and Day 2 (d-f) for D0C, D1C and D2C respectively. ‘Other’ was categorised as less than 1% of bacterial OTUs among the population. Bacteria at baseline or at Day 2 were not significant different between intervention sessions. D0C = Meal with no spices intervention, D1C = Meal with low (6 g) mixed spices intervention, D2C = Meal with high (12 g) mixed spices intervention, *UG* unknown genus, *UF* unknown family. n = 15 each intervention.

**Figure 2:**
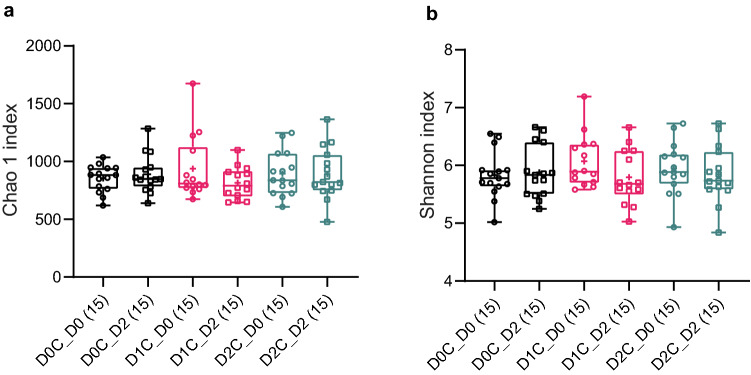
Alpha diversity of the three different doses of spices intervention (**a**) Chao 1’s and (**b**) Shannon’s indexes were measured and described at Y axis. No significant difference between spices intervention were found by Friedman’s test with Dunn’s multiple comparison within Day 0 or Day 2. The types and days of intervention were drawn as different symbols and colours. + shows mean. D0C = Meal with no spices intervention, D1C = Meal with low (6 g) mixed spices intervention, D2C = Meal with high (12 g) mixed spices intervention. No of samples were in the parenthesis.

There were no significant differences in the relative abundances of the individual bacterial genera at baseline (Day 0) or at Day 2 as calculated using Friedman’s test with Dunn’s multiple comparison as shown in Fig. 1 and Supplementary Tables S1 and S2. The alpha diversity of each spices intervention is shown in Fig. 2. No significant differences were found between the various intervention sessions for both the Chao 1 and Shannon diversity indices.

**Figure 3:**
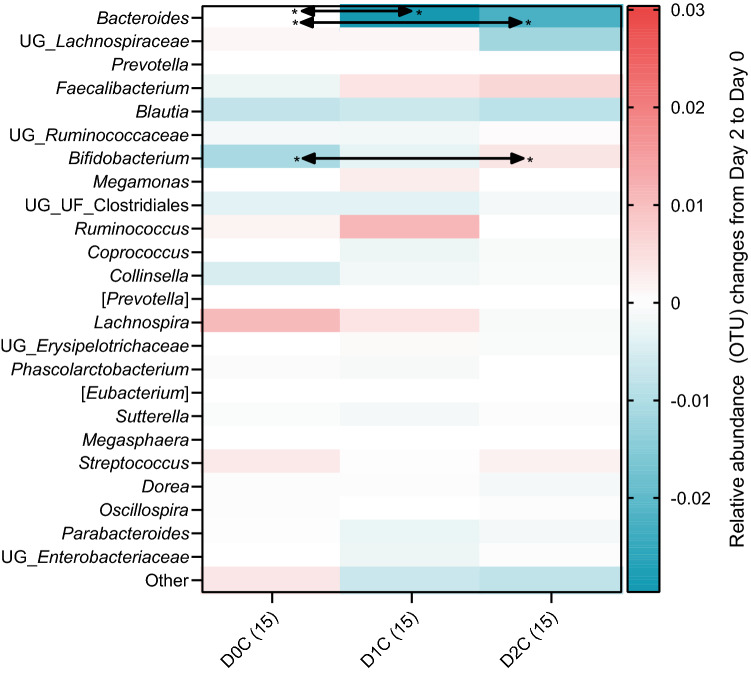
Heatmap of medians relative abundance changes of the major bacterial genera after two days of intervention for the three doses of mixed spices intervention. ‘Other’ was categorised as less than 1% of bacterial OTUs among the population. The relative abundance changes (Day 2 minus Day 0) in bacteria between various doses were analysed using Friedman’s test with Dunn’s multiple comparison and described as * adjusted p < 0.05.

However, the spice intervention led to significant changes in the relative abundances of two bacterial genera. The changes from Day 0 to Day 2 in the relative abundance of the various bacterial genus (Operational Taxonomic Units: OTUs) during each of the three intervention sessions are shown as a heatmap in Fig. 3.

**Figure 4:**
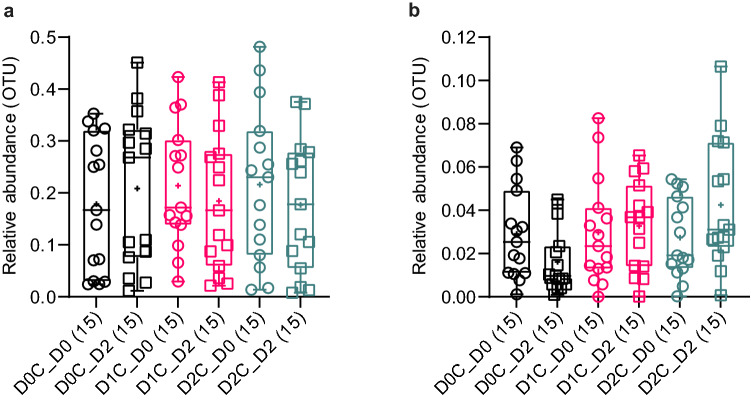
Relative abundance distribution of (**a**) *Bacteroides* and (**b**) *Bifidobacterium* at Day 0 and Day 2 for three doses of mixed spices intervention. No bacteria were significantly different between three different doses of spices intervention analysed by Friedman’s test with Dunn’s multiple comparison. D0C = Meal with no spices intervention, D1C = Meal with low (6 g) mixed spices intervention, D2C = Meal with high (12 g) mixed spices intervention. *UG* unknown genus, *UF* unknown family. No of samples (each intervention) was 15 and described in the parenthesis.

**Figure 5:**
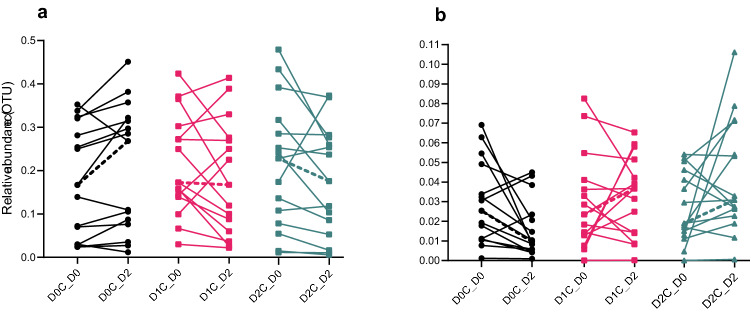
Paired individual changes in relative abundance between two time points (Day 0 to Day 2) for three spices doses (**a**) *Bacteroides* and (**b**) *Bifidobacterium*. Wilcoxon matched-pairs signed rank test was applied and significant pairs were described as * p < 0.05. D0C = Meal with no spices intervention, D1C = Meal with low (6 g) mixed spices intervention, D2C = Meal with high (12 g) mixed spices intervention. n = 15 each intervention. Dashed line indicates median change.

As can be seen in the heatmap (Fig. 3), compared with the control, the relative abundance of *Bacteroides* was significantly reduced with increasing spice doses (adjusted p < 0.05 for both D0C vs D1C and D0C vs D2C). This was mainly driven by the fact that there was an increase in *Bacteroides* relative abundance changes from Day 0 to Day 2 during the D0C session, whereas the *Bacteroides* relative abundance from Day 0 to Day 2 either remained the same or decreased slightly during D1C and D2C sessions. On the contrary, the relative abundances of *Bifidobacterium* (adjusted p < 0.05 for D0C vs D2C) increased significantly with spice intake as compared with control. This was also driven by the fact that there was a decrease in *Bifidobacterium* relative abundance changes from Day 0 to Day 2 during the D0C session, whereas the *Bifidobacterium* relative abundance from Day 0 to Day 2 either remained the same or increased slightly during D1C and D2C sessions. The extent and the variabilities in the relative abundances in these two genera during the various intervention sessions at Day 0 and 2 are shown in Fig. 4 as median and inter-quartile range (IQR). The paired individual changes in relative abundances between two time points (Day 0 to Day 2) for three spices doses in these two genera is shown in Fig. 5.

**Figure 6:**
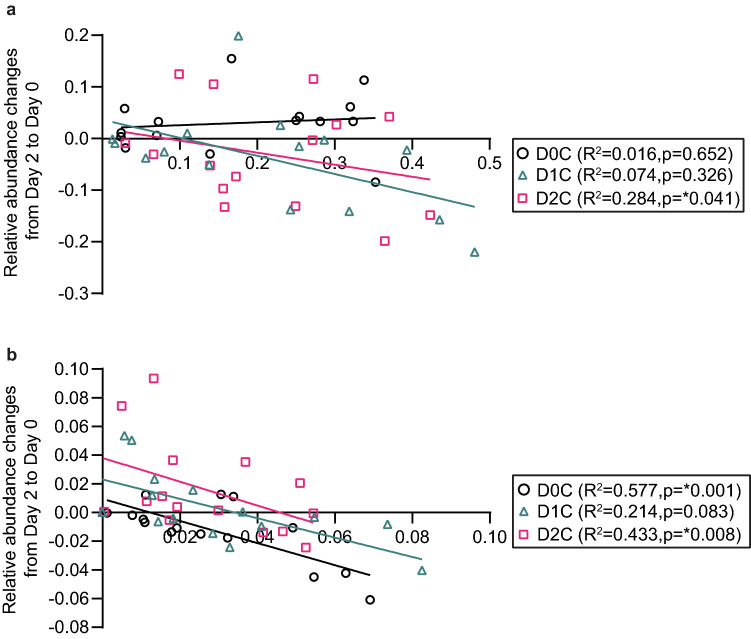
Changes in relative abundances of (**a**) Bacteroides and (**b**) Bifidobacterium in relation to baseline relative abundance for the three doses. Simple linear regression was applied and R^2^ and p values were described for the group that had *p < 0.05. The relative abundance of bacteria at Day 0 and relative abundance changes were represented at X and Y axes, respectively. The colours represent the respective spices intervention session. D0C = Meal with no spices intervention, D1C = Meal with low (6 g) mixed spices intervention, D2C = Meal with high (12 g) mixed spices intervention. No of samples (each intervention) was 15.

Figure 6 shows the changes in relative abundances of (a) *Bacteroides* and (b) *Bifidobacterium* in relation to their absolute relative abundance at baseline (Day 0) for all three doses. We found a significant correlation (R^2^ = 0.284, p = 0.041) for D2C in *Bacteroides* but, no such correlation for D0C. On the other hand, those with low *Bifidobacterium* relative abundance at baseline had the greatest changes in relative abundances for D0C (R^2^ = 0.577, p = 0.001) and D2C (R^2^ = 0.433, p = 0.008).

### Association between changes of bacterial genus and two plasma phenolic acids and changes urinary total polyphenol excretion in 24 h

As shown in Table 2, when we pooled the data across all intervention time points, we found significant positive associations between plasma concentration (on Day 1) of CNA and *Bifidobacterium* whereas negative associations between plasma CNA and *Bacteroides*, *Ruminococcus* and *Oscillospira*. Similarly, plasma PAA concentration was also negatively associated with *Bacteroides, Lachnospira* and *Phascolarctobacterium.* On the contrary, only *Coprococcus*, had a significant negative association with changes in 24 h urinary total polyphenol excretion (Day 1–Day 0).

**Table 2:** The correlation between changes in 24 h urinary total polyphenol excretion, plasma cinnamic acid AUC_0–7 h,_ and plasma phenyl acetic acid AUC_0–7 h_ and relative abundance changes of the bacterial genus from Day 0 to Day 2 of mixed spices intervention, for all intervention doses combined.

	Changes in urinary total polyphenol excretion (Day 1 minus Day 0)		Plasma cinnamic acid AUC_0–7 h Day 1_		Plasma phenyl acetic acid AUC_0–7 h Day 1_	
	Spearman r	p values (two-tailed)	Spearman r	p values (two-tailed)	Spearman r	p values (two-tailed)
*Bacteroides*	− 0.173	0.224	− 0.300	*0.038	− 0.299	*0.033
UG_*Lachnospiraceae*	0.104	0.467	− 0.097	0.512	− 0.052	0.717
*Prevotella*	0.186	0.192	0.075	0.613	0.015	0.914
*Faecalibacterium*	0.098	0.493	− 0.189	0.199	0.133	0.354
*Blautia*	− 0.260	0.065	0.013	0.929	− 0.055	0.703
UG_*Ruminococcaceae*	− 0.050	0.725	− 0.019	0.900	0.187	0.189
*Bifidobacterium*	0.076	0.598	0.372	**0.009	0.222	0.117
*Megamonas*	0.137	0.339	0.183	0.214	− 0.053	0.714
UG_UF_*Clostridiales*	− 0.220	0.121	0.179	0.224	− 0.063	0.661
*Ruminococcus*	− 0.007	0.961	− 0.308	*0.033	− 0.016	0.909
*Coprococcus*	− 0.298	*0.034	0.122	0.411	0.153	0.283
*Collinsella*	− 0.033	0.818	0.269	0.065	0.255	0.071
*[Prevotella]*	0.065	0.649	− 0.254	0.082	0.181	0.205
*Lachnospira*	0.167	0.243	− 0.255	0.081	− 0.284	*0.043
UG_*Erysipelotrichaceae*	− 0.128	0.370	− 0.087	0.556	− 0.194	0.173
*Phascolarctobacterium*	− 0.205	0.149	− 0.136	0.355	− 0.354	*0.011
*[Eubacterium]*	− 0.231	0.102	− 0.146	0.322	0.062	0.667
*Sutterella*	− 0.032	0.825	0.272	0.061	− 0.022	0.879
*Megasphaera*	0.133	0.353	0.226	0.122	0.192	0.177
*Streptococcus*	− 0.140	0.329	0.112	0.449	− 0.194	0.173
*Dorea*	− 0.255	0.071	− 0.239	0.101	− 0.068	0.635
*Oscillospira*	− 0.062	0.664	− 0.288	*0.047	0.112	0.435
*Parabacteroides*	− 0.068	0.637	− 0.153	0.298	0.044	0.761
UG_*Enterobacteriaceae*	− 0.004	0.978	0.029	0.843	0.096	0.504
Other (< 1% of total bacteria)	− 0.165	0.248	− 0.141	0.340	0.089	0.533

Non-parametric Spearman correlation coefficient (r) and two-tailed p values were shown.

*AUC* area under curve, *UG* unknown genus, *UF* unknown family. n = 15 each intervention.

**p ≥ 0.001– < 0.01, *p < 0.05.

## Discussion

While polyphenolic compounds in diet are known to modulate gut microbiome^16–18^, metabolism of dietary polyphenols also rely on the gut microbiome to exert their biological effects in humans, particularly for their breakdown into secondary metabolites and subsequent absorption^31^. This bi-directional association between dietary polyphenols and the gut microbiome is integral to the host-gut bacteria symbiosis. The present study is one of the few studies in humans investigating the influence of polyphenol-rich mixed spices, contained within curry meals, in two separate doses on acute changes in gut microflora. To the best of our knowledge, there has been only one other similar previous study undertaken with mixed spices in humans^27^. While this previous study by Lu et al. explored the effects of mixed spices consumption (5 g/day) over a longer time frame (2 weeks), our study showed that even a single meal containing mixed spices can modify/restore gut microflora within a period of 24–48 h. Moreover, our study had a rigorous study design of avoiding polyphenol-rich foods 3-days prior to each intervention session (i.e., during run-in period) and the rest of the meals during the measurement days apart from the intervention (D0C, D1C, and D2C) test meals were standardized and provided. Given that our study was undertaken in a Chinese population who otherwise consumed Indian mixed spices less frequently, it minimised any residual confounding due to prior habituation of mixed spices intake. It appears from our results that a continuous adherence of a low polyphenol diet during the D0C (control) session led to an increase in the relative abundance of *Bacteroides* and a decrease in the relative abundance of *Bifidobacterium* between Days 0 to 2 of the intervention, whereas the re-introduction of polyphenol rich spices at the two doses of curry somewhat restored/reversed this trend within this same time frame. The rapid time frame of this change in gut microbiome as a result of a single bout of curry, at 2 separate doses, is not surprising given that previous studies have also shown alterations in gut microflora within a 24-h time scale^32–34^.

Prior to their human trial, Lu et al. undertook a separate study in vitro which showed prebiotic potential, altering greater than 120 bacterial species, including the growth promotion of certain probiotics such as *Bifidobacterium* spp. and inhibition of growth of pathogenic bacteria such as *Clostridium* spp.^35^. In their human trial, the same authors found that the mixed spices affected 26 OTUs, including growth promotions of *Bifidobacteria *spp.*, Lactobacilli *spp.*, Bacteroides *spp. and inhibition of *Clostridia* spp.^27^. In our trial, while *Bifidobacterium* also increased in the highest dose of mixed spices (D2C) vs control (D0C), there were significant reductions in *Bacteroides* (in both D0C and D2C vs D0C) populations with increasing mixed spices dose. The variations in the findings between the two studies may be due to differences in the background diet, the initial bacterial populations and/or differences in host genetics. *Prevotella* and *Bacteroides* are the two main genera of the same *Bacteroidetes* phylum and are highly abundant in human stool samples^36^. It is generally accepted that vegetable-rich diets tend to give rise to a greater abundance of *Prevotella* whereas animal-based diets tend to be enriched in *Bacteroides*^37,38^. Similarly, vegetarians and vegans have greater *Prevotella/Bacteroides* ratio than Omnivore populations^39,40^, as well as rural populations having a greater *Prevotella/Bacteroides* ratio as compared with urban populations^34,41^. However, we did not find any differences in *Prevotella/Bacteroides* ratio in our study most likely due to dietary fibre in the above-mentioned dietary patterns driving such effects.

Indeed, previous studies found that increasing the intake of various polyphenols, such as dealcoholized red wine extracts rich in resveratrol^42^ and pomegranate rich extracts^43^ led to increases in *Prevotella* populations, although we did not observe this effect within our study population. In support of our study, a previous study reported that in vitro incubation of dietary polyphenolic components such as chlorogenic acid, caffeic acid, and rutin reduced the *Bacteroides* population under anaerobic conditions^44^. In vivo studies in rats also found a decrease in *Bacteroides* population upon feeding of polyphenol-rich cocoa^45^. Even within our lab, we have shown that tea phenolic metabolites can extensively suppress the growth of *Bacteroides*^22^. Thus, taking into account these previous findings, the modulation of *Bacteroides* by polyphenol-rich mixed spices observed in our present study is likely to be causally associated and further studies are needed to confirm this. It should also be noted that the median reduction in relative abundance of *Bacteroides* was driven by a subset of voulnteers (Fig. 5a) who mainly had higher level of this bacteria at Day 0 as discussed in detail below and presented in Fig. 6a. This is further supported by no differences in the median *Bacteroides* relative abundance between various spice dose at Day 2 (nor Day 0 as expected, as shown in Fig. 5a). The negative associations between both CNA and PAA with *Bacteroides* further reiterates the decreases in the relative abundance of this bacteria with increasing doses of mixed spice rich curries may well be causally linked, as we have previously shown that the concentrations of both CNA and PAA could be used as objective biomarkers of the intake of the mixed spices used in our study^30^.

Several previous studies have indicated that polyphenol-rich foods can increase the abundance of *Bifidobacterium*^46^. Among the randomized controlled trials, one study with dealcoholized red wine showed significant increases in *Bifidobacterium*^47^, which was in fact further associated with increases in secondary metabolites of anthocyanins including several phenolic acids^48^. Similarly, consumption of wild blueberries^49^ as well as tart cherries^50^ were also shown to increase *Bifidobacteria*. Furthermore, the study by Lu et al.^27^ with five mixed spices, mentioned earlier, also found increases in the abundance of several *Bifidobacteria spp*. This, together with the findings of our study of the increases in *Bifidobacteria* with the highest dose of spice (D2C) meal indicates that polyphenol-rich spices may have bifidogenic properties. Hence, our study supports the concept of polyphenols having prebiotic properties, as discussed previously^51^. However, it should also be noted that the median increase in relative abundance of *Bifidobacterium* at higher dose of spices was mainly driven by only a subset of voulnteers, with low relative abundance at baseline (as shown in Fig. 5b), as discussed in more detail below (and shown in Fig. 6b). This is further supported by no differences in the median *Bifidobacterium* relative abundance between various spice dose at Day 2 (nor Day 0 as expected, as shown in Fig. 5b). Nonetheless, the positive association between plasma CNA and *Bifidobacteria* could also be a causal effect, since various hydroxycinnamic acids such as chlorogenic acid and caffeic acid have been previously shown to stimulate the growth of *Bifidobacteria*^44^.

In our study, the consumption of curry meals did not alter the alpha diversity of the gut microbiome of the tested subjects (Fig. 1). This may be due to the acute nature of the intervention as well as human gut microbiome, particularly the core members which account for most of the abundance in the gut microbiome that has been found to possess a high functional response diversity through functionally genetic redundancy^52^. This allows them to adapt well to short-term changes in the diet, which could explain why there were rarely large-scale changes in gut microbiota profile despite daily and seasonal variation in dietary ingredients and preparation^53,54^. Despite this adaptive capability, we have shown that a single bout of mixed spices can lead to, or restore compared to control dose (D0C session) significant modulation of certain gut microbes, particularly at the higher dose of curry (i.e., D2C meal). However, it remains to be established whether similar changes occur following longer-term consumption of polyphenol-rich spices and whether such changes are stable over time. Indeed this is one of the main limitations of this study in that finding reported here only describes acute modifications in gut microbiome. Another limitation of this study is that we only looked at associations of gut microbial changes with specific targeted metabolites in plasma/urine. Therefore future studies with individual and/or mixed spices should not only be undertaken with a longer duration of dietary intervention but should also explore how the changes in gut microbiome may be resulting from global changes in the metabolome in plasma and/or stool samples, arising from the intake of specific foods and/or food groups, in order to determine known/unknown metabolites that may directly influence certain bacterial populations. Nonetheless, with the globalization of dietary habits and the increasing amount of spices being consumed globally, the data from our study indicate an opportunity for bringing about beneficial changes in the gut microbiome through the intake of polyphenol-rich spices. Moreover, characterization of the gut microbiome across various parts of the world and various ethnic groups and/or cultures is an expanding area of research^43,55^ and further work needs to be done on the extent to which specific diet and lifestyle practices can modulate microbiome within these populations. Importantly, the functional consequences of the modulation of the microbiota, including what constitutes a ‘healthy microbiome’ are yet to be fully established^56,57^. Furthermore, recent reviews highlighted the utility of these polyphenol-associated gut microbiota changes on several anthropometric and clinical parameters related to cardiometabolic health^18,58,59^ and therefore changes in gut microbiome may be one of the major mechanisms through which dietary polyphenols may exert their widely reported benefits. The findings in our study also support these previous reports that some downstream metabolites of dietary polyphenols present in the mixed spices such as CNA and PAA may be directly influencing the bacterial changes observed with the intake of mixed spices used in our study.

Finally, the relative abundance at baseline of both *Bacteroides* and *Bifidobacterium* influenced the extent to which the changes in relative abundances of these two bacteria occurred at the various doses of curry. The regression plot in Fig. 6a shows a greater decrease in the relative abundance in *Bacteroides* in those individuals with a greater baseline relative abundance in this bacteria, particularly for Dose 2 curry, although this association was not present during the Dose 0 control session. Similarly, the regression plot in Fig. 6b shows that those with a lower relative abundance of *Bifidobacterium* had the greatest increase in the relative abundance (positive change) of the bacteria between Day 0 and Day 2 for both Dose 1 curry and Dose 2 curry. In comparison, majority of the individuals during Dose 0 control either had a reduction (negative change) in the *Bifidobacterium* relative abundance or did not change. This further highlights that the effects of dietary components on gut bacteria are likely to be dependent on the individual microbiome profile and hence may explain the inter-individual differences in responses to various dietary interventions.

## Conclusions

This dose–response study has shown that even a single dose of mixed spices curry can modify/restore gut microbiome as compared with a control diet which was low in polyphyenols, within a 24–48-h timeframe. While the overall alpha-diversity of gut microbiome did not change as a result of this dietary intervention, there were in fact decrease/restoration in *Bacteroides* populations with increasing mixed spices doses relative to the dose 0 control (D0C) diet, when there was an increase in this bacteria. Similarly, our study also showed an increase/restoration in *Bifidobacteria* populations in the highest curry dose (D2C) compared with control (D0C), which further supports the bifidogenic potential of polyphenols. It should also be noted that the relative abundance changes in both *Bacteroides* and *Bifidobacterium* observed within each individual dose of spices (D0C, D1C and D2C) were mainly driven by a subset of individuals (Figs. 4 and 5), indicating large intra and inter-individual variabilities in responses to the dietary intervention. The reasons for this variability remains to be additionally investigated and was beyond the remit of this study. Furthermore, longer term studies are required to confirm our acute findings and a more detailed array of metabolites should be measured in order to establish mechanisms responsible for such changes.

## Methods

### Dietary intervention design

The study protocol, including the inclusion and exclusion criteria, has been detailed elsewhere^15^. In brief, the study was a randomized, crossover, acute, food based intervention trial specifically undertaken in 21–40-year-old, healthy, Chinese men, with a BMI between 18.5 and 27.5 kg/m^2^, who would otherwise typically not consume large amounts of Indian spices as part of their habitual diet. This secondary analysis was only undertaken in a subset of 15 volunteers who provided stool samples for the study and have completed all three doses of the dietary intervention. A per-protocol approach was utilized and data from three volunteers were excluded. These volunteers did not complete all 3 doses of intervention since we used matching analyses across all 3 doses. The study was approved by the Domain Specific Research Board (DSRB) ethics committee, Singapore (Reference: C/2015/00729) and was registered at clinicaltrials.gov (ID: NCT02599272) and was undertaken in accordance with the Declaration of Helsinki, revised in 2013 and as per Singapore Good Clinical Practice Guidelines. Informed consents from all volunteers were obtained before the intervention study.

Each volunteer in the study undertook 3 intervention sessions which were completed in random order which was obtained using an online randomization generator (http://randomizer.org). These sessions included a Dose 0 Control (D0C, no spices) session, Dose 1 Curry (D1C, low spices) session, and the Dose 2 Curry (D2C, high spices) session. The test meals for the D1C and D2C sessions included a mixture of 7 dried spice powders at doses 6 g and 12 g respectively. The spice mix was made up of turmeric, cumin, coriander, *amla* (Indian gooseberry), cinnamon, clove, and cayenne pepper mixed in the ratios of 8:4:4:4:2:1:1 respectively. The individual polyphenols present in the ingredients used to prepare the various curries (D1C and D2C) or the control (D0C) are listed in Supplementary Table S3.

The schematics of the study design is shown in Supplementary Fig. S1. In the 3 days prior to the main study day (Day 1) when the mixed spice containing meals (D1C or D2C) or the control meal (D0C) were consumed, all participants were asked to avoid consumption of any spice or any other polyphenol-rich foods (i.e., the ‘run-in period’). To aid their compliance, a list of common foods rich in polyphenols and common spices were provided to them. Furthermore, a 3-day food diary was completed during the ‘run-in period’ for each study session to record all foods that they consumed to further facilitate the detection of erroneous spices or polyphenol-rich food consumption during these periods and to improve compliance to the dietary intervention. One day before the main study day of each session, the volunteers were required to provide their baseline (Day 0) stool sample prior to the food based intervention on Day 1. On the main study day (Day 1), in the morning after an overnight fast, the volunteer consumed one of 3 intervention meals (D0C, D1C or D2C test meals). The total energy, macronutrients as well as total vegetable contents of the 3 intervention meals were comparable as reported previously^15^. The rest of the meals and snacks on Day 1 were standardized and contained low amounts of polyphenol containing ingredients and no spices within them. On the following day (Day 2), another stool sample was provided. Therefore, Day 0 stool represented the gut microbiome of the participants prior to each intervention session, after abstaining from polyphenol-rich foods and spices for the previous three days, whereas, Day 2 stool sample represented the gut microbiome immediately after each dietary intervention. Between each study session, each participant had at least a 14-day ‘washout period’ during which the participants resumed their habitual diet. This was done to avoid any ‘carry-over’ effect from one intervention session to the next.

### Polyphenol analyses in urine and plasma

Urine samples were also collected over 2 × 24 h periods between Day 0 to Day 2, in 2 × 3 L plastic containers (Simport, Canada), to measure total polyphenol excretion (TPE) using the Folin-Ciocalteu assay as described in detail previously^29^. ‘Day 0’ urine was collected in the first urine container from the morning of Day 0 morning until Day 1 morning immediately prior to the consumption of test meals. ‘Day 1’ urine was collected during the subsequent 24 h period immediately after the test meal consumption between Day 1 morning until Day 2 morning. Blood samples were also collected in K_2_ EDTA vacutainer tubes (BD, Franklin Lakes, NJ, USA) immediately after the consumption of test meals on Day 1, at regular intervals (12 time points) to measure concentrations of various phenolic acids in plasma using a UHPLC-MS/MS method, as described in detail previously^30^. The areas under the curve (AUC) of postprandial concentrations of plasma phenolic acids were calculated using the trapezoid method.

### Stool sample collection and 16 s rRNA gene sequencing analysis

For each stool sample collection (Day 0 and Day 2), the volunteers were asked to provide approximately 5 g of stool sample which was dissolved in a universal tube containing 2 ml of RNAlater solution (Ambion, Inc., Texas, USA) and were analysed for gut microbiota profiling with 16 s rRNA gene sequencing. To do this, 0.2 ml of faecal homogenate from approximately 1 g of collected stool was extracted using the phenol–chloroform method after washing with Phosphate-buffered saline (PBS). Along with the glass-beads mechanical extraction, Tris-SDS, TE-saturated phenol (Sigma-Aldrich, Cor., St.Louis, Missouri, USA) and phenol/chloroform/isoamyl alcohol (25:24:1) solutions were used. DNA was precipitated with sodium acetate and isopropanol followed by washing with ethanol and eluted in TE. The quantified DNA was normalised to 12.5 ng and amplified with a primer set that targeted at the regions of v3 and v4 of the 16 s rRNA gene. Once the amplicons were purified with Agencourt AMPure XP beads (Beckman Coulter, Inc., Fullerton, CA, USA), they were amplified with the Nextera XT index primers (Illumina, Inc., San Diego, USA). The quantified and normalised library was denatured and spiked with the PhiX control library followed by sequencing in the Miseq system (Illumina, Inc., San Diego, USA).

### Bioinformatics and statistical analyses

The 16 s rRNA gene sequence outputs were analysed with Quantitative Insights Into Microbial Ecology (QIIME) version 1.9.1^60^. After joining the reads and quality filtering, the chimeric sequences were removed with USEARCH v6.1^61^. The non-chimeric sequences were picked to get the operational taxonomic unit (OTU) using 97% similarity sequences of Greengenes v13_8 database. The assigned OTUs were summarised into the taxonomical category for the bacterial profiling of samples in three intervention sessions. Chao 1 and Shannon diversity indexes of alpha diversity were computed using the OTU table. The 3-day food diary data were converted into the average energy and nutrients values per day by an online tool of energy and nutrient composition of food Health Promotion Board, Singapore^62^.

The bacterial genus taxonomy data, alpha diversity data and the dietary nutrients data were further, analysed and normality tests were performed. Bacterial genus with more than 1% of the relative abundance of the total bacterial population were included in this study and 24 major bacterial genera were found. Mean and Standard Deviation (SD) were described for the average energy and nutrients such as energy (kcal), protein (g), total fats (g), saturated fat (g), fibre (g), carbohydrate (g), cholesterol (mg) and sodium (mg) per day. Friedman’s test with Dunn’s multiple comparison tests were compared the different intervention groups at baseline (Day 0). The consumption of the dietary energy and nutrients were analysed with one-way ANOVA mixed-effects analysis. For comparison of changes of major bacteria, abundances of *Bacteroides* and *Bifidobacterium* and Alpha diversity from Day 2 to Day 0 for the three spices intervention groups, Friedman with Dunn’s multiple comparison tests were performed. Paired individual changes of above two bacteria were analysed by Wilcoxon matched-pairs signed rank test. A non-parametric Spearman rho and two-tailed p values were calculated for the correlation between changes of three metabolites and relative abundance of bacterial genera. The changes in the relative abundances of two bacteria (*Bacteroides* and *Bifidobacterium*) in relation to their absolute abundance at baseline (Day 0) for the various curry doses (D0C, D1C and D2C) were analysed using simple linear regression. The comparison of relative abundance of major bacteria at baseline were provided and described as Mean ± SD and the intra-individual variation at baseline between the three doses is presented as mean % Coefficient of Variation (%CV). All the statistical analyses were performed by GraphPad Prism 8 (GraphPad Software Inc., San Diego, USA).

## Supplementary Information

Supplementary Information 1.

Supplementary Information 2.

## Data Availability

The relevant data are provided in the paper and the raw sequencing data can be found at the EBI repository (accession no: PRJEB35853).
