# Supplementary material for: A single serving of mixed spices alters gut microflora composition: a dose–response randomised trial
Source: Sci Rep. 2021 May 28;11:11264. doi: 10.1038/s41598-021-90453-7 (PMC8163817; doi:10.1038/s41598-021-90453-7)
Supplement: Supplementary file 2 — Supplementary Information 2. [file 41598_2021_90453_MOESM2_ESM.docx]

A single serving of mixed spices alters gut microflora composition: a dose-response randomised trial

Wei Wei Thwe Khine, Sumanto Haldar, Shou De Loi, Yuan-Kun Lee

Correspondence: Yuan-Kun Lee

**Supplementary Information**

**Supplementary Figure S1: Study design**. Adapted from Haldar et al. *Eur J Nutr.* 58:301-313 (2019). Each volunteer completed 3 separate dietary intervention sessions in random sequence, with a minimum of 1 week between sessions, consisting of Dose 0 Control (D0C), Dose 1 Curry (D1C) and Dose 2 Curry (D2C) consumed for breakfast on Day 1. SM, standardized meal; SS, standardized snacks provided at other times were the same for all 3 intervention sessions. Urine samples were collected for 24 h periods on Day 0 and Day 1. Blood samples were collected for 7 h following Test Meal breakfast on Day 1. Stool samples were collected on Day 0 and Day 2. (attached separately: Supplementary Figure S1.eps)

**Supplementary Table S1: Relative abundance of bacterial genera for the three different doses of spices intervention at baseline (Day 0)**

**Supplementary Table S2: Relative abundance of bacterial genera for the three different doses of spices intervention at Day 2**

**Supplementary Table S3: List of various polyphenols found in the individual ingredients used to prepare the test meals (Dose 0 Control, Dose 1 Curry and Dose 2 Curry)**. Adapted from Haldar et al. *Nutrients*. 10, 934 (2018). Polyphenol components were obtained using from Phenol-Explorer 3.6 database (http://phenol-explorer.eu/ , accessed April 2018)

**Supplementary Dataset: Results of relative abundance of the bacterial genus** (attached separately: Supplementary Dataset.xlsx)

**

**

**Supplementary Figure S1: Study design**. Adapted from Haldar et al. *Eur J Nutr.* 58:301-313 (2019). Each volunteer completed 3 separate dietary intervention sessions in random sequence, with a minimum of 1 week between sessions, consisting of Dose 0 Control (D0C), Dose 1 Curry (D1C) and Dose 2 Curry (D2C) consumed for breakfast on Day 1. SM, standardized meal; SS, standardized snacks provided at other times were the same for all 3 intervention sessions. Urine samples were collected for 24 h periods on Day 0 and Day 1. Blood samples were collected for 7 h following Test Meal breakfast on Day 1. Stool samples were collected on Day 0 and Day 2.

**Supplementary Table S1: Relative abundance of bacterial genera for the three different doses of spices intervention at baseline (Day 0)**

| Bacteria | Mean ± SD | | | Mean of %CV | Adjusted P value |
| --- | --- | --- | --- | --- | --- |
|  | D0C (n=15) | D1C (n=15) | D2C (n=15) |  |  |
| *Bacteroides* | 0.178 ± 0.13 | 0.214 ± 0.118 | 0.216 ± 0.149 | 31.897 | 0.78 |
| UG_*Lachnospiraceae* | 0.155 ± 0.062 | 0.14 ± 0.058 | 0.148 ± 0.06 | 18.508 | >0.999 |
| *Prevotella* | 0.154 ± 0.188 | 0.095 ± 0.135 | 0.117 ± 0.157 | 58.707 | >0.999 |
| *Faecalibacterium* | 0.078 ± 0.039 | 0.083 ± 0.046 | 0.071 ± 0.04 | 39.05359 | >0.999 |
| *Blautia* | 0.083 ± 0.033 | 0.075 ± 0.023 | 0.079 ± 0.03 | 21.50499 | >0.999 |
| UG_*Ruminococcaceae* | 0.038 ± 0.024 | 0.044 ± 0.035 | 0.04 ± 0.026 | 33.57341 | >0.999 |
| *Bifidobacterium* | 0.029 ± 0.021 | 0.03 ± 0.025 | 0.027 ± 0.018 | 59.33482 | >0.999 |
| *Megamonas* | 0.023 ± 0.039 | 0.022 ± 0.026 | 0.024 ± 0.025 | 75.16646 | >0.999 |
| UG_UF_Clostridiales | 0.022 ± 0.009 | 0.022 ± 0.009 | 0.022 ± 0.009 | 21.36051 | >0.999 |
| *Ruminococcus* | 0.013 ± 0.016 | 0.014 ± 0.015 | 0.017 ± 0.018 | 55.25757 | >0.999 |
| *Coprococcus* | 0.015 ± 0.008 | 0.018 ± 0.012 | 0.015 ± 0.007 | 28.06525 | >0.999 |
| *Collinsella* | 0.017 ± 0.013 | 0.025 ± 0.032 | 0.014 ± 0.01 | 44.59233 | >0.999 |
| *[Prevotella]* | 0.015 ± 0.028 | 0.015 ± 0.038 | 0.021 ± 0.044 | 68.92521 | >0.999 |
| *Lachnospira* | 0.009 ± 0.007 | 0.011 ± 0.01 | 0.015 ± 0.012 | 59.23893 | >0.999 |
| UG_*Erysipelotrichaceae* | 0.013 ± 0.013 | 0.014 ± 0.012 | 0.012 ± 0.013 | 43.3871 | >0.999 |
| *Phascolarctobacterium* | 0.011 ± 0.01 | 0.014 ± 0.015 | 0.011 ± 0.011 | 50.66159 | >0.999 |
| *[Eubacterium]* | 0.013 ± 0.022 | 0.01 ± 0.012 | 0.014 ± 0.02 | 65.6982 | >0.999 |
| *Sutterella* | 0.011 ± 0.007 | 0.011 ± 0.006 | 0.011 ± 0.008 | 31.11046 | >0.999 |
| *Megasphaera* | 0.011 ± 0.015 | 0.01 ± 0.013 | 0.011 ± 0.014 | 72.33274 | >0.999 |
| *Streptococcus* | 0.009 ± 0.009 | 0.008 ± 0.009 | 0.011 ± 0.013 | 70.47688 | >0.999 |
| *Dorea* | 0.009 ± 0.005 | 0.011 ± 0.009 | 0.01 ± 0.006 | 31.72381 | >0.999 |
| *Oscillospira* | 0.006 ± 0.006 | 0.009 ± 0.009 | 0.007 ± 0.005 | 53.91619 | >0.999 |
| *Parabacteroides* | 0.005 ± 0.006 | 0.008 ± 0.007 | 0.008 ± 0.01 | 58.64488 | >0.999 |
| UG_*Enterobacteriaceae* | 0.008 ± 0.011 | 0.018 ± 0.039 | 0.005 ± 0.007 | 107.7727 | >0.999 |
| Other | 0.075 ± 0.035 | 0.08 ± 0.036 | 0.071 ± 0.018 | 30.44925 | >0.999 |

Differences ~~between~~ across the three doses (adjusted p values) were calculated using Friedman test with Dunn’s multiple comparison. Intra-individual variation for each bacterial genus at baseline were calculated as the mean of individual %CV across three doses. Other was categorised as less than 1% of bacterial OTUs among the population. D0C = Meal with no spices intervention, D1C =Meal with low (6g) mixed spices intervention, D2C = Meal with high (12g) mixed spices intervention, UG= Unknown genus, UF= Unknown family. SD= Standard Deviation, CV= Coefficient of Variation. n= 15 each intervention

**Supplementary Table S2: Relative abundance of bacterial genera for the three different doses of spices intervention at baseline (Day 2)**

| Bacteria | Mean ± SD | | | Mean of %CV | Adjusted P value |
| --- | --- | --- | --- | --- | --- |
|  | D0C (n=15) | D1C (n=15) | D2C (n=15) |  |  |
| *Bacteroides* | 0.209 ± 0.148 | 0.184 ± 0.133 | 0.177 ± 0.128 | 37.896 | >0.999 |
| UG_*Lachnospiraceae* | 0.156 ± 0.052 | 0.143 ± 0.071 | 0.145 ± 0.058 | 21.687 | >0.999 |
| *Prevotella* | 0.144 ± 0.168 | 0.138 ± 0.188 | 0.147 ± 0.191 | 47.341 | >0.999 |
| *Faecalibacterium* | 0.076 ± 0.042 | 0.084 ± 0.063 | 0.084 ± 0.049 | 36.910 | >0.999 |
| *Blautia* | 0.073 ± 0.03 | 0.069 ± 0.04 | 0.068 ± 0.031 | 33.632 | >0.999 |
| UG_*Ruminococcaceae* | 0.04 ± 0.026 | 0.043 ± 0.036 | 0.044 ± 0.031 | 28.595 | >0.999 |
| *Bifidobacterium* | 0.016 ± 0.015 | 0.033 ± 0.021 | 0.042 ± 0.029 | 66.708 | 0.56 |
| *Megamonas* | 0.02 ± 0.021 | 0.039 ± 0.053 | 0.03 ± 0.037 | 79.837 | >0.999 |
| UG_UF_Clostridiales | 0.019 ± 0.008 | 0.017 ± 0.008 | 0.021 ± 0.008 | 24.933 | >0.999 |
| *Ruminococcus* | 0.02 ± 0.018 | 0.023 ± 0.021 | 0.016 ± 0.018 | 50.500 | >0.999 |
| *Coprococcus* | 0.017 ± 0.016 | 0.018 ± 0.015 | 0.015 ± 0.011 | 23.870 | >0.999 |
| *Collinsella* | 0.01 ± 0.007 | 0.016 ± 0.015 | 0.014 ± 0.01 | 43.582 | 0.428 |
| *[Prevotella]* | 0.015 ± 0.029 | 0.013 ± 0.025 | 0.018 ± 0.036 | 83.757 | >0.999 |
| *Lachnospira* | 0.021 ± 0.013 | 0.018 ± 0.016 | 0.019 ± 0.02 | 52.207 | >0.999 |
| UG_*Erysipelotrichaceae* | 0.014 ± 0.017 | 0.013 ± 0.012 | 0.012 ± 0.013 | 48.178 | >0.999 |
| *Phascolarctobacterium* | 0.011 ± 0.008 | 0.011 ± 0.01 | 0.013 ± 0.013 | 45.445 | >0.999 |
| *[Eubacterium]* | 0.01 ± 0.016 | 0.012 ± 0.021 | 0.012 ± 0.018 | 63.750 | >0.999 |
| *Sutterella* | 0.011 ± 0.007 | 0.01 ± 0.007 | 0.01 ± 0.004 | 31.683 | >0.999 |
| *Megasphaera* | 0.007 ± 0.007 | 0.01 ± 0.014 | 0.012 ± 0.013 | 69.716 | 0.655 |
| *Streptococcus* | 0.012 ± 0.013 | 0.01 ± 0.009 | 0.013 ± 0.016 | 55.539 | >0.999 |
| *Dorea* | 0.012 ± 0.014 | 0.009 ± 0.005 | 0.008 ± 0.005 | 38.233 | >0.999 |
| *Oscillospira* | 0.006 ± 0.003 | 0.006 ± 0.005 | 0.006 ± 0.006 | 52.079 | >0.999 |
| *Parabacteroides* | 0.005 ± 0.004 | 0.005 ± 0.006 | 0.005 ± 0.005 | 47.604 | >0.999 |
| UG_*Enterobacteriaceae* | 0.002 ± 0.001 | 0.005 ± 0.007 | 0.003 ± 0.005 | 85.221 | >0.999 |
| Other | 0.075 ± 0.031 | 0.071 ± 0.032 | 0.067 ± 0.032 | 31.394 | >0.999 |

Differences across the three doses (adjusted p values) were calculated using Friedman test with Dunn’s multiple comparison. Intra-individual variation for each bacterial genus at baseline were calculated as the mean of individual %CV across three doses. Other was categorised as less than 1% of bacterial OTUs among the population. D0C = Meal with no spices intervention, D1C =Meal with low (6g) mixed spices intervention, D2C = Meal with high (12g) mixed spices intervention, UG= Unknown genus, UF= Unknown family. SD= Standard Deviation, CV= Coefficient of Variation. n= 15 each intervention

**Supplementary Table S3: List of various polyphenols found in the individual ingredients used to prepare the test meals (Dose 0 Control, Dose 1 Curry and Dose 2 Curry)**. Adapted from Haldar et al. *Nutrients*. 10, 934 (2018). Polyphenol components were obtained using from Phenol-Explorer 3.6 database (http://phenol-explorer.eu/ , accessed April 2018)

| Food | Food form | Polyphenol class | Polyphenol sub-class | Polyphenol |
| --- | --- | --- | --- | --- |
| Turmeric | Turmeric dried | Other polyphenols | Curcuminoids | Bisdemethoxycurcumin |
|  |  |  |  | Curcumin |
|  |  |  |  | Demethoxycurcumin |
|  |  |  |  |  |
| Cumin | Cumin | Flavonoids | Flavonols | Kaempferol |
|  |  |  | Flavanones | Eriodictyol |
|  |  |  | Flavones | Luteolin |
|  |  | Phenolic acids | Hydroxycinnamic acid | Caffeic acid |
|  |  |  |  | Ferulic acid |
|  |  |  | Hydroxybenzoic acids | Gallic acid |
|  |  |  |  |  |
| Coriander | Coriander, seed |  | |  |
| Cinnamon | Ceylan cinnamon | Phenolic acids | Hydroxybenzoic acids | 2-Hydroxybenzoic acid |
|  |  |  |  | Protocatechuic acid |
|  |  |  |  | Syringic acid |
|  |  |  | Hydroxycinnamic acids | Caffeic acid |
|  |  |  |  | p-Coumaric acid |
|  |  |  |  |  |
| Clove | Cloves | Flavonoids | Flavonols | Kaempferol |
|  |  |  |  | Quercetin |
|  |  | Other polyphenols | Hydroxyphenylpropenes | Acetyl eugenol |
|  |  |  |  | Eugenol |
|  |  | Phenolic acids | Hydroxybenzoic acids | Gallic acid |
|  |  |  |  | Protocatechuic acid |
|  |  |  |  | Syringic acid |
|  |  |  |  | p-Coumaric acid |
|  |  |  |  |  |
| Cayenne pepper | Cayenne pepper, raw | Flavonoids | Flavones | Luteolin |
|  |  |  | Flavonols | Quercetin |
| Gooseberry (amla) | Gooseberry | Flavonoids | Anthocyanins | Cyanidin 3-O-(6"-caffeoyl-glucoside) |
|  |  |  |  | Cyanidin 3-O-(6"-p-coumaroyl-glucoside) |
|  |  |  |  | Cyanidin 3-O-galactoside |
|  |  |  |  | Cyanidin 3-O-glucoside |
|  |  |  |  | Cyanidin 3-O-rutinoside |
|  |  |  |  | Peonidin 3-O-glucoside |
|  |  |  |  | Peonidin 3-O-rutinoside |
|  |  |  | Flavonols | (+)-Catechin |
|  |  |  |  | (+)-Gallocatechin |
|  |  |  |  | Kaempferol |
|  |  |  |  | Quercetin |
|  |  | Lignans | Lignans | Secoisolariciresinol |
|  |  | Phenolic acids | Hydroxybenzoic acids | 4-Hydroxybenzoic acid 4-O-glucoside |
|  |  |  |  | Protocatechuic acid 4-O-glucoside |
|  |  |  | Hydroxycinnamic acids | 3-Caffeoylquinic acid |
|  |  |  |  | 3-Feruloylquinic acid |
|  |  |  |  | 3-p-Coumaroylquinnic acid |
|  |  |  |  | Caffeic acid 4-O-glucoside |
|  |  |  |  | Caffeoyl glucose |
|  |  |  |  | Ferulic acid 4-O-glucoside |
|  |  |  |  | p-Coumaric acid 4-O-glucoside |
|  |  |  |  | p-Coumaroyl glucose |
|  |  |  |  |  |
| Garlic | Garlic fresh | Lignans | Lignans | Lariciresinol |
|  |  |  |  | Matairesinol |
|  |  |  |  | Pinoresinol |
|  |  |  |  | Secoisolariciresinol |
|  |  |  |  |  |
| Ginger | Ginger, fresh | Lignans | Lignans | Secoisolariciresinol |
|  |  |  |  |  |
| Onion | Onion (red), raw | Flavonoids | Anthocyanins | Cyanidin 3-O-(6"-malonyl-3"-glucosyl-glucoside) |
|  |  |  |  | Cyanidin 3-O-(6"malonyl-glucoside) |
|  |  |  |  | Delphinidin 3-O-glucosyl-glucoside |
|  |  |  | Flavonols | Isorhamnetin |
|  |  |  |  | Isorhamnetin 4'-O-glucoside |
|  |  |  |  | Quercetin |
|  |  |  |  | Quercetin 3,4'-O-diglucoside |
|  |  |  |  | Quercetin 3-O-glucoside |
|  |  |  |  | Quercetin 3-O-rutinoside |
|  |  |  |  | Quercetin 4'-O-glucoside |
|  |  |  |  | Quercetin 7,4'-O-diglucoside |
|  |  |  |  | Kaempferol |
|  |  |  |  | Myricetin |
|  |  |  | Flavones | Apigenin |
|  |  |  |  | Luteolin |
|  |  | Phenolic acids | Hydroxybenzoic acids | Protocatechuic acid |
|  |  |  |  |  |
| Tomato | Tomato, whole, raw | Flavonoids | Flavanones | Naringenin 7-O-glucoside |
|  |  |  |  | Naringenin |
|  |  |  | Flavonols | Kaempferol |
|  |  |  |  | Quercetin |
|  |  |  |  | Quercetin 3-O-rutinoside |
|  |  |  |  | Myricetin |
|  |  |  | Flavones | Apigenin |
|  |  | Lignans | Lignans | Lariciresinol |
|  |  |  |  | Matairesinol |
|  |  |  |  | Medioresinol |
|  |  |  |  | Pinoresinol |
|  |  |  |  | Secoisolariciresinol |
|  |  |  |  | Syringaresinol |
|  |  | Phenolic acids | Hydroxycinnamic acids | 4-Caffeoylquinic acid |
|  |  |  |  | 5-Caffeoylquinic acid |
|  |  |  |  | Caffeic acid |
|  |  |  |  | Ferulic acid |
|  |  |  |  | p-Coumaric acid |
|  |  |  | Hydroxybenzoic acids | 4-Hydroxybenzoic acid |
|  |  |  |  | Vanillic acid |
|  |  |  |  |  |
| Aubergine | Aubergine, peeled | Phenolic acid | Hydroxybenzoic acids | 4-Hydroxybenzoic acid |
|  |  |  |  | Gallic acid |
|  |  |  |  | Protocatechuic acid |
|  |  |  | Hydroxycinnamic acids | Caffeic acid |
|  |  |  |  | Ferulic acid |
|  |  |  |  | p-Coumaric acid |
